# Thermodynamic and Economic Analyses of Reformative Design for High Back-Pressure Heating in Coal-Fueled Cogeneration Units

**DOI:** 10.3390/e21040342

**Published:** 2019-03-28

**Authors:** Heng Chen, Yunyun Wu, Jidong Xu, Gang Xu, Yongping Yang, Wenyi Liu, Gangye Shi

**Affiliations:** 1 National Thermal Power Engineering and Technology Research Center, North China Electric Power University, Beijing 102206, China; 2 East China Electric Power Design Institute, Shanghai 200063, China

**Keywords:** combined heat and power, heating system, high back pressure, energy cascade utilization, thermodynamic analysis, economic analysis

## Abstract

High back-pressure (HBP) heating technology has been identified as an effective approach to improve the efficiency of combined heat and power (CHP). In this study, the novel concept of a HBP heating system with energy cascade utilization is developed and its probability examined. In the reformative design, the extracted heating steam from the intermediate-pressure turbine (IPT) is first drawn to an additional turbine where its excess pressure can be converted into electricity, then steam with a lower pressure can be employed to heat the supply water. As a consequence, the exergy destruction in the supply water heating process can be reduced and the efficiency of the cogeneration unit raised. A detailed thermodynamic investigation was performed based on a typical coal-fired HBP–CHP unit incorporating the proposed configuration. The results show that the artificial thermal efficiency (ATE) promotion was as much as 2.01 percentage points, with an additional net power output of 8.4 MW compared to the reference unit. This was attributed to a 14.65 percentage-point increment in the exergy efficiency of the supply water heating process caused by the suggested retrofitting. The influences of the unit power output, unit heat output, supply water and return water temperatures and turbine back pressure on the thermal performance of the modified system are discussed as well. In addition, the economic performance of the new design is assessed, indicating that the proposed concept is financially feasible.

## 1. Introduction

Combined heat and power (CHP) cogeneration is a method of generating power and heat in parallel from one energy source at or near the site of consumption, and its potential to provide benefits comes from making the best possible utilization of fuel energy [1,2] which can convert the chemically bound fuel energy into electricity and heat at an overall efficiency above 90% [3,4]. Cogeneration is also capable of reducing fuel consumption by 20–30% as compared to the decoupled production in conventional power and heating stations [5,6]. Furthermore, by recovering and using heat, cogeneration units can diminish environmental emissions in the generation sector by 13–18% [7], which has been recognized as playing a crucial role in energy sustainability and the attempt to achieve climate change response objectives [8,9]. Since cogeneration can alleviate the need for increased power generation capacity and reduce the environmental impact associated with energy consumption, it has been broadly utilized in different countries [10,11,12]. In China, the operational CHP capacity has been over 300 million kW since 2016, and 350 million kW of coal-fired power units will be retrofitted into cogeneration units for district heating before 2020 [13]. It is obvious that cogeneration will become more significant for district heating globally.

In a conventional large-scale coal-fired cogeneration unit, the supply water of the primary heating network is heated by the extraction steam (ES) from the steam turbine [14], whose pressure and temperature are about 0.3–0.5 MPa and 235–276 °C, respectively. Due to climate change and energy-efficient building technologies, the supply water temperature of the primary loop has declined from 120–130 °C to below 100 °C at present [15,16]. Consequently, the extraction steam pressure and temperature could be much higher than the requested values in consideration of the decreased supply water temperature, and severe exergy destruction may be induced during the practical heat exchange between the extraction steam and supply water. Therefore, the energy utilization of the traditional heating system is inadequate, and its amelioration is necessary.

Aiming at enhancing the thermal performance of CHP, a volume of research has been carried out on advanced heating technologies, mainly including absorption heat pump (AHP), absorption heat exchanger (AHE) and high back-pressure (HBP) heating [17]. Both AHP and AHE can recover the waste heat of exhaust steam (ET) via working fluids such as H_2_O–LiBr and NH_3_–H_2_O [18,19]. A number of authors have reported on the energy-saving effects and sensitivities of AHP [20,21,22] and AHE [23,24]. As a promising way to promote the CHP efficiency at a low cost, the HBP heating technology has attracted much attention over the past few years. Its adoption is encouraged in retrofitting power generation plants into cogeneration by the Chinese government [25]. The thermodynamic characteristics of the HBP heating system have been explored by several researchers [13,26,27,28]. However, there is still a massive loss of exergy in a typical HB–CHP unit due to the pressure waste of the extracted heating steam, which must be reduced to further boost unit efficiency.

Against this backdrop, a hybrid design combining cascade heating and waste pressure utilization for HBP–CHP units has been put forward. The overall performance of the optimized scheme was evaluated from the perspectives of thermodynamics and economics. Energy and exergy analyses were conducted with regard to the proposed system, and the root cause of energy-saving as a result of the new design was revealed. The impacts of the unit power output, the unit heat output, the heating water temperatures and the turbine back pressure were also comprehensively considered under different conditions. Finally, the economic benefit of the proposed concept was investigated and its feasibility was further verified. The objective of the present paper is to modify the heating system of the cogeneration unit and conserve the fuel consumed for power production and district heating.

## 2. System Description

### 2.1. Regular HBP Heating System

**Figure 1:**
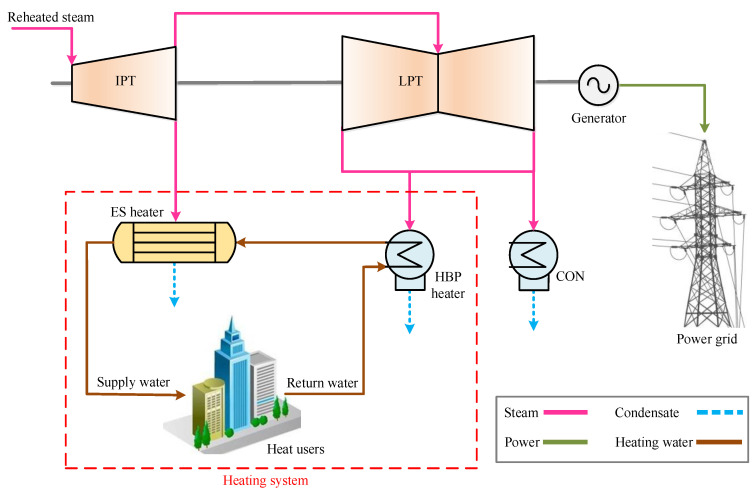
Regular HBP heating system in a coal-fueled cogeneration unit. (IPT, intermediate-pressure turbine; LPT, low-pressure turbine; HBP, high back-pressure; CON, condenser; ES, extraction steam).

Typically, the heating system based on a large-scale HBP steam turbine is mainly composed of an HBP heater and an ES heater, as illustrated in Figure 1. The exhaust steam pressure of the low-pressure turbine (LPT) is usually as high as 34–54 kPa, and the corresponding saturation temperature is approximately 72–83 °C. Firstly, the supply water goes through the HBP heater and obtains energy from the exhaust steam. The supply water temperature can reach 70–81 °C at the outlet of the HBP heater if the upper-terminal temperature difference of the HBP heater remains above 2 °C. If the supply water of 70–81 °C is hot enough, it can be conveyed to consumers via the primary loop directly. When the weather is cold, the supply water will be heated to a higher temperature in the ES heater by the extraction steam from the intermediate-pressure turbine (IPT), and the outlet water temperature is usually 80–100 °C. Hence, the HBP heating system can recover the waste heat of the exhaust steam and conserve the extraction steam for heating, which can dramatically promote the overall efficiency of the cogeneration unit. However, plenty of exergy is still lost in the ES heater, because the extraction steam pressure is much higher than the required value and the temperature difference is relatively high during the heat exchange. Moreover, the excessive steam pressure is completely wasted, which could be utilized to do work. It seems that the regular HBP heating system needs to be optimized and the energy of the extraction steam should be employed more fully.

### 2.2. Proposed HBP Heating System

**Figure 2:**
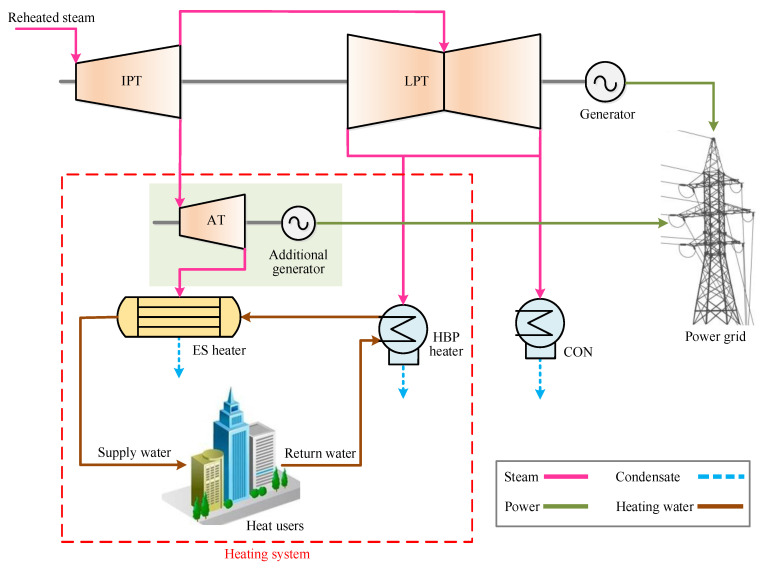
Proposed HBP heating system in a coal-fueled cogeneration unit.

To reduce the exergy destruction in the supply water heating process, a novel HBP heating design with energy cascade utilization has been brought forward, as shown in Figure 2. The extracted heating steam is first exploited to drive the additional turbine (AT) and additional generator for generating electricity. Afterwards, the steam pressure will fall from 0.3–0.5 MPa to about 0.1 MPa or lower, guaranteeing that the upper-terminal temperature difference of the ES heater is above 10 °C, namely, maintaining the steam saturation temperature 10 °C larger than the outlet supply water temperature. The electricity exported from the additional generator can be used as auxiliary power on site, or transmitted to power consumers through the power grid. By converting the excess steam pressure into electricity, the exergy destruction caused by the pressure disuse can be diminished, which implies that the new configuration takes advantage of the steam energy more sufficiently and conduces to the advancement of the cogeneration unit. The proposed concept was applied in the reference unit and its thermo-economic performance and sensitivity were comparatively explored.

## 3. System Simulation and Evaluation Criteria

### 3.1. Reference HBP–CHP Unit

**Figure 3:**
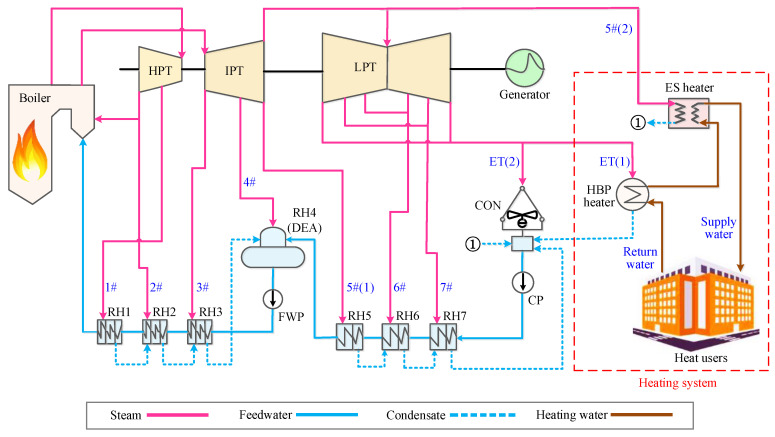
Diagram of the reference HBP–CHP unit. (CP, condensate pump; ET, exhaust steam; RH, regenerative heater; HPT, high-pressure turbine; CHP, combined heat and power; DEA, deaerator; FWP, feedwater pump).

The reference case is a subcritical coal-fired cogeneration unit cooperating an HBP heating system, located in the north of China. As Figure 3 depicts, the steam turbine consists of a high-pressure turbine (HPT), an IPT and two LPTs associated with an air-cooling condenser (CON). There are three high-pressure regenerative heaters (RH), one deaerator (DEA) and three low-pressure RHs installed in the feedwater cycle. The data of the reference HBP–CHP unit under the rated heating condition are displayed in Table 1 and Table 2. The heating service of this unit lasts from 15 November to 15 March of the next year, and the space-heating demand of 6.52 million square meters in a rural area can be satisfied. During the heating season, the back pressure of the LPT increases from 14 kPa to 34 kPa and the HBP heater is adopted to heat the supply water of the primary loop. Then, the supply water flows into the ES heater and gains energy from the stage 5# extraction steam. While the back pressure is 34 kPa, the supply water can be heated up to 70 °C by the HBP heater using the exhaust steam of about 72 °C. On the condition that the weather is relatively warm and the required supply water temperature is below 70 °C, the ES heater does not work. If necessary, the supply water can be heated up to 80–100 °C by the ES heater. Under the rated heating condition, the theoretical lowest steam pressure for sufficiently heating in the ES heater is approximately 0.09 MPa (when the supply water temperature is 86 °C and the upper-terminal temperature difference of the heater is regarded as 10 °C for efficient heat exchange, the saturation pressure is about 0.09 MPa, corresponding to the saturation temperature of 96 °C), which implies that the actual extraction steam pressure of 0.39 MPa is much higher than the requisite value, leading to a high-temperature difference and much exergy destruction in the actual heat exchange process. It is essential to make the most of the excess steam pressure and minimize the exergy loss.

### 3.2. Model Development and Verification

Simulations of the cogeneration unit were performed on Ebsilon Professional software, which specializes in the design, simulation and optimization of thermodynamic cycles in power systems [29]. The software contains an abundant database that can be used for directly calculating the parameters of a power system at various conditions with high degrees of fidelity [30]. With reference to the design data of the case unit, the simulation model of the cogeneration unit without/with the optimized heating design was built according to the balances of mass and energy, and the model details are introduced in Table 3.

The availability of the established model was examined by comparing the calculated values with the design values that are provided by the manufactures of the boiler and turbine. For the reference case, the calculation errors of the unit gross power output under the conditions of turbine heat acceptance (THA), as well as 75%, 50%, 40% and 30% THA, are presented in Table 4. These results indicate that the maximum relative error was only 0.52%, and that the simulation model is accurate and reliable. The off-design operation data of the reference and proposed units were derived on the basis of the built model.

### 3.3. Thermal Evaluation Criteria

#### 3.3.1. Energy Efficiency

Because of the difficulties in quantifying the benefits achieved by the usage of CHP over a traditional system, many appraisal criteria have been developed [31]. As two common measures, the energy utilization factor (*EUF*) and artificial thermal efficiency (*ATE*) were adopted to assess the cogeneration units based on the first thermodynamic principle.

The energy utilization factor is expressed as Equation (1), which has the advantage of simplicity. It is a measure for comparing the efficiency of a CHP system to that of a conventional supply, which is helpful in understanding the benefits of CHP. Nevertheless, *EUF* cannot discriminate between $P_{\mathrm{net}}$ and $Q_{h}$ from the perspective of energy level, implying that *EUF* is not a satisfactory criterion for CHP [32].

(1) $$EUF=\frac{P_{\mathrm{net}}+Q_{h}}{Q_{f}}$$

where $Q_{f}$ donates the total energy of fuel input (MW); $P_{\mathrm{net}}$ donates the net power output of the unit (MW); $Q_{h}$ donates the net heat output of the unit (MW).

An alternative criterion for energy performance of cogeneration units is artificial thermal efficiency, which is given by Equation (2) [33]. Artificial thermal efficiency offers a direct performance comparison between CHP and a conventional power generation system, considering the qualities of heat and electricity.

(2) $$ATE=\frac{P_{\mathrm{net}}}{Q_{f}-Q_{h}/\eta_{b,\mathrm{ref}}}$$

where $\eta_{b,\mathrm{ref}}$ donates the efficiency of the reference “heat only” boiler, which was chosen as 0.83 for a coal-fired boiler [34].

In addition, the waste heat of the exhaust steam is recovered by the HBP design, and making use of more exhaust steam contributes to a higher energy efficiency of the cogeneration unit [28]. The recovery efficiency of the exhaust steam ($\eta_{\mathrm{rec}}$) is defined as Equation (3), which is another performance indicator of the HBP configuration.

(3) $$\eta_{\mathrm{rec}}=\frac{m_{\text{et-h}}}{m_{\mathrm{et}}}$$

where $m_{\mathrm{et}}$ donates the total exhaust steam flow rate (t/h); $m_{\text{et-h}}$ donates the exhaust steam flow rate into the HBP heater (t/h).

#### 3.3.2. Exergy Efficiency

Exergy is a measure of the maximum work that can be generated from a certain amount of energy or material [35]. Exergy analysis is favorable for improving the efficiency of energy-recourse usage because it quantifies the locations, types and magnitudes of wastes and losses, which accurately identifies the margins available for devising more-efficient energy systems through reducing inefficiencies [36,37]. The exergy of one steady matter flow (without regard to chemically bonded exergy) can be calculated as

(4) $$ex_{m}=h_{m}-h_{0}-T_{0}\times (s_{m}-s_{0})$$

(5) $$EX_{m}=ex_{m}\times m_{m}/10^{3}$$

where $ex_{m}$ donates the specific exergy of the matter flow (kJ/kg); $h_{m}$ and $h_{0}$ donate the specific enthalpies of the matter flow at the present state and environmental state (kJ/kg), respectively; $T_{0}$ donates the environmental temperature (K); $s_{m}$ and $s_{0}$ donate the specific entropies of the matter flow at the present state and environmental state (kJ/kgK), respectively; $EX_{m}$ donates the total exergy of the matter flow (MW); $m_{m}$ donates the flow rate of the matter flow (kg/s).

The exergy efficiency of the heating process ($\eta_{\mathrm{ex}}$) was taken to assess the performance of the proposed design on grounds of the second thermodynamic law, which is defined as follows [38]:

(6) $$\eta_{\mathrm{ex}}=\frac{EX_{\mathrm{cold},\mathrm{out}}-EX_{\mathrm{cold},\mathrm{in}}}{EX_{\mathrm{hot},\mathrm{in}}-EX_{\mathrm{hot},\mathrm{out}}}$$

where $EX_{\mathrm{cold},\mathrm{in}}$ and $EX_{\mathrm{cold},\mathrm{out}}$ donate the total inlet exergy and outlet exergy of the cold fluid (MW), respectively; $EX_{\mathrm{hot},\mathrm{in}}$ and $EX_{\mathrm{hot},\mathrm{out}}$ donate the total inlet exergy and outlet exergy of the hot fluid (MW), respectively.

## 4. Results and Discussion

### 4.1. Overall Performance

The proposed scheme was thermodynamically and economically evaluated compared to the conventional one, and several assumptions have been made:

- the coal consumption rate of the cogeneration unit is maintained constant;
- the pressures and temperatures of the main steam and reheated steam are kept identical;
- the boiler efficiency is fixed
- the heat output of the cogeneration unit and the supply water and return water temperatures remain unchanged;
- the influence of surrounding on the cogeneration unit is ignored.

**Figure 4:**
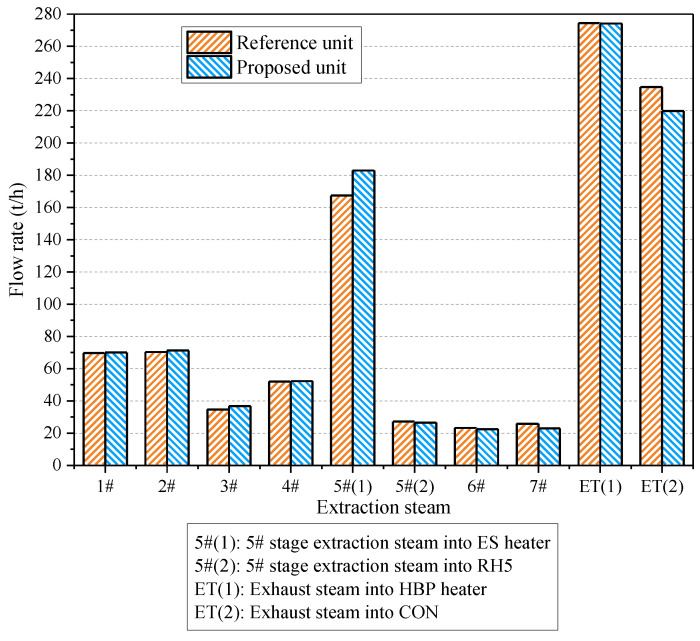
Extraction steam flow rates in the reference and proposed units.

The overall performances of the proposed HBP–CHP unit and the reference are presented in Table 5. The steam pressure into the ES heater dwindles from 0.39 to 0.09 MPa after the retrofitting, and the excess pressure of the extracted heating steam is converted into electricity, which contributes to an additional net power output of 8.4 MW for the CHP system. In consequence, the energy utilization factor and artificial thermal efficiency are promoted by 1.08 and 2.01 percentage points, respectively. Aside from this, the specific enthalpy of the extracted heating steam does not decline much through the AT in the reformative scheme, implying that most of the energy of the extraction steam still releases into the ES heater. Table 5 and Figure 4 indicate that, in contrast to the reference unit, more extraction steam (5#(1)) from the IPT is tapped in the modified one, which can expand in the AT to generate electricity, and the steam conveyed into the LTP is reduced accordingly. Therefore, less exhaust steam gets into the CON, and energy loss to the ambient is cut down, while the recovery efficiency of the exhaust steam is raised from 53.90% to 55.49%. Above all, the new design can not only employ the waste pressure of the extracted heating steam, but also diminish the energy loss of the exhaust steam. These findings confirm that the reformative heating configuration is capable of remarkably boosting the performance of the cogeneration unit, and plenty of economic benefits may be expected.

### 4.2. Energy and Exergy Analyses

**Figure 5:**
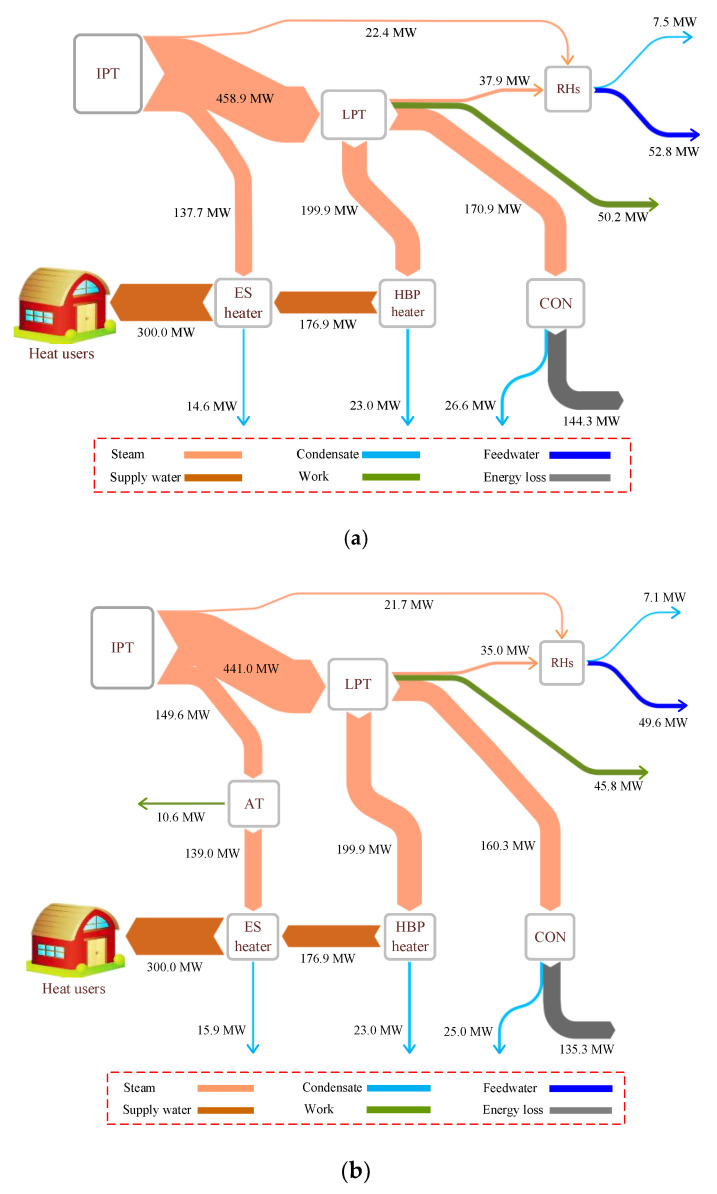
Energy flow diagrams of the reference and proposed heating systems: (**a**) reference system and (**b**) proposed system. (AT, additional turbine).

Figure 5 illustrates the energy flows that take place in the two HBP heating systems. The heat absorbed by the supply water in the HBP heater and the ES heater remains unchanged after the retrofitting. However, more energy is obtained from the IPT along with the extraction steam for heating in the reformative configuration, and 10.6 MW energy of the extraction steam is converted into work through the AT. At the same time, the waste heat released from the CON is reduced by 9.0 MW as a result of the new design, contributing to a decrease in the total energy loss of the unit, and furthermore, the work output of the LPT declines by 4.4 MW, because less steam gets access to the LPT from the IPT in the modified scheme.

**Figure 6:**
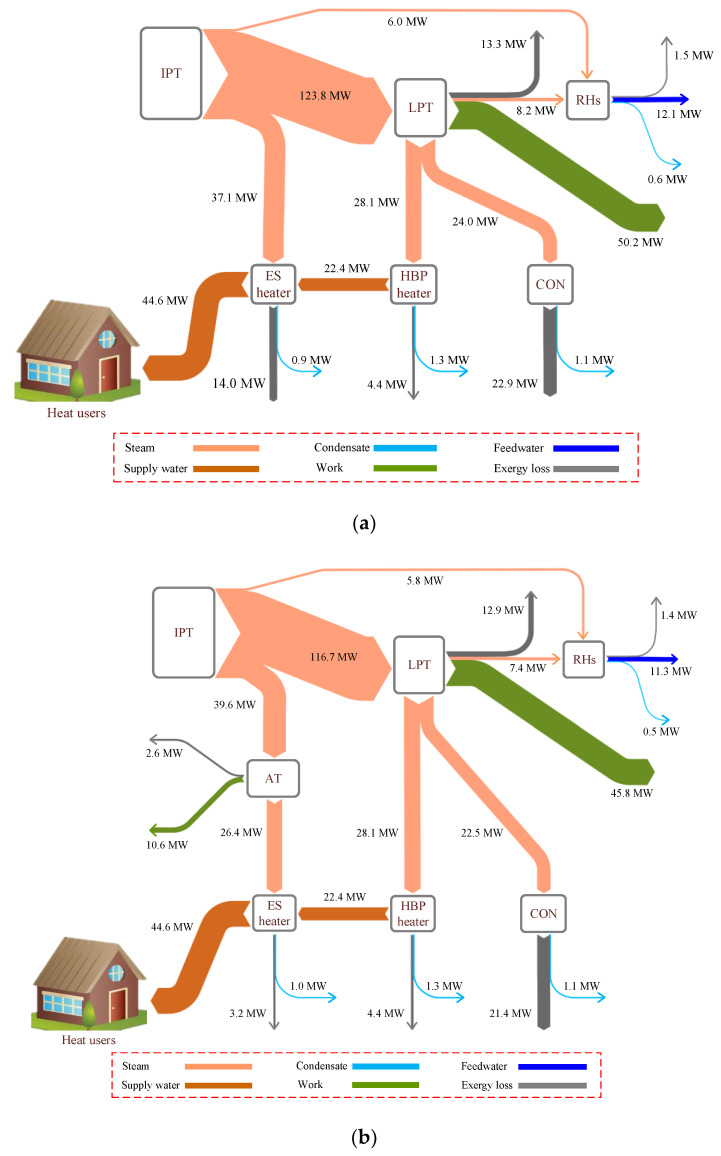
Exergy flow diagrams of the reference and proposed heating systems: (**a**) reference system and (**b**) proposed system.

To gain insights into the energy conservation mechanism of the proposed concept, the exergy flows of the two HBP heating systems were drawn, as shown in Figure 6. The exergy destruction in the ES heater of the conventional system is 10.8 MW more than that of the retrofitted one, because the temperature difference during the heat exchange is large and the superfluous extraction steam pressure is completely wasted. However, the new design reduces the exergy destruction by converting the excess pressure of the extraction steam into work via the AT, and the temperature difference between the hot and cold fluids declines. Hence, the exergy destruction in the ES heater falls from 14.0 MW to 3.2 MW, with an extra work output of 10.6 MW. Owing to the suggested optimization, the exergy destructions in the LPT and CON diminish as well, because less steam is sent into the LPT and CON.

**Figure 7:**
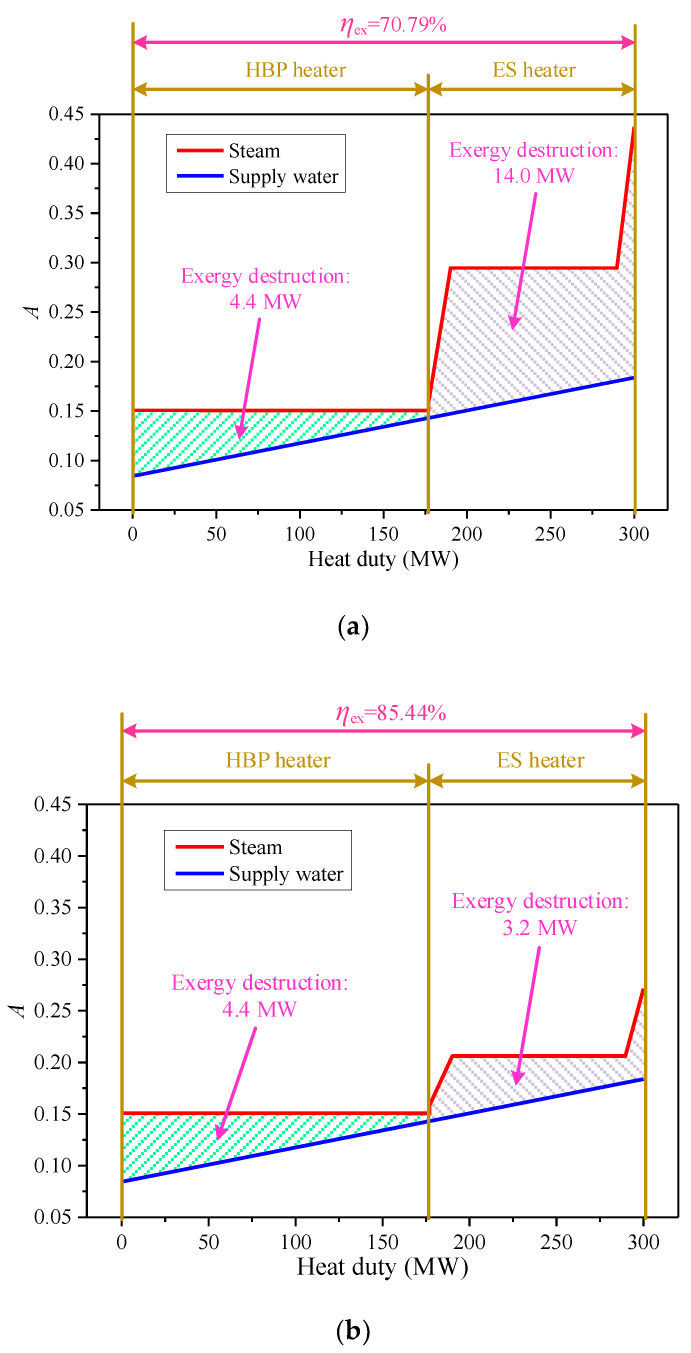
Energy utilization diagram analyses of the reference and proposed supply water heating processes: (**a**) reference process and (**b**) proposed process.

For the purpose of exploring the inherent exergy change during the supply water heating, the energy utilization diagram (EUD) analysis was carried out on the heating process, as displayed in Figure 7, and the energy level *A* was calculated as Equation (7) [39,40]. It is obvious that the exergy destruction in the ES heater drops dramatically, because the steam saturation temperature decreases from 142.7 °C to 96.7 °C due to the new design, and *A* remarkably dwindles accordingly. As a consequence, the exergy efficiency of the supply water heating process is improved from 70.79% to 85.44%, which is beneficial to the advancement of the cogeneration unit.

(7) $$A=1-\frac{T_{0}\times \Delta S}{\Delta Q}$$

where ΔS and ΔQ donate the entropy change and energy change during the heat transfer process, respectively.

### 4.3. Parametric Analysis

#### 4.3.1. Effect of Unit Power Output

**Figure 8:**
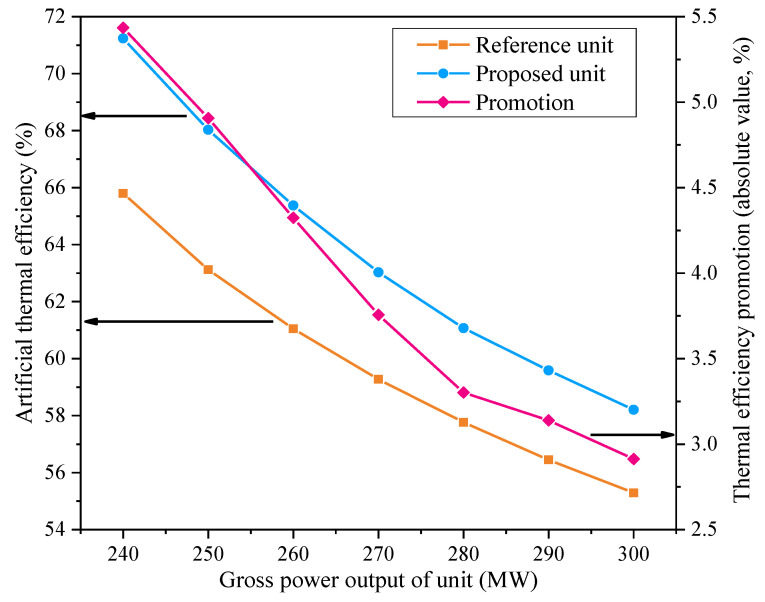
Influences of the unit power output on the performances of the reference and proposed units.

Figure 8 illustrates the impact of the unit power output on the thermal efficiency promotion caused by the proposed concept. When the cogeneration unit generates less electricity under the heat output of 300.0 MW, the optimized design contributes to a larger increment in the artificial thermal efficiency; thereby, more fuel is conserved. As the unit power output rises, the ratio of the extracted heating steam to the main steam falls and the effective impact of the modified configuration recedes. In the north of China, the generation capacities of cogeneration units may be severely restricted at low levels in winter, to leave more room for renewable power (for example, wind power, solar power and so on); nevertheless, the heating demand is huge [41]. Furthermore, the total installed power capacity is also superfluous in these regions, facing an oversupply potential of electricity [42]. It seems that these cogeneration units usually operate at low-power outputs, but during the heating season they operate at high-heat outputs. Therefore, the new design can play a more significant role in the energy conservation of the cogeneration units located in northern China.

#### 4.3.2. Effect of Unit Heat Output

**Figure 9:**
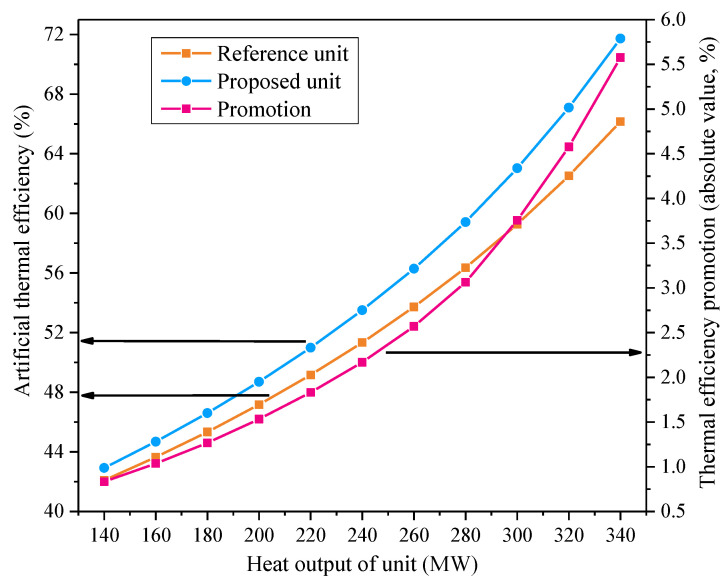
Influences of the unit heat output on the performances of the reference and proposed units.

The relationship between the unit heat output and the energy-saving benefit of the reformative system is displayed in Figure 9. If the unit heat output gets larger, the artificial thermal efficiencies of the two units noticeably rise. One reason for this could be that the higher the unit heat output is, the more waste heat of the exhaust steam will be recovered by imparting energy to the supply water, promoting unit efficiency. The power output of the additional generator is positively correlated with the extraction steam for heating, which is enhanced when the unit heat output grows. As a consequence, thermal efficiency promotion due to the suggested retrofitting increases with the rise of the unit heat output under a constant unit power output, and more excess pressure of the extraction stem can be converted into electricity, diminishing the steam pressure damage. It is likely that the modified configuration tends to save more fuel under high heat outputs and the artificial thermal efficiency improvement may reach up to above 5.5 percentage points.

#### 4.3.3. Effect of Supply Water and Return Water Temperatures

**Figure 10:**
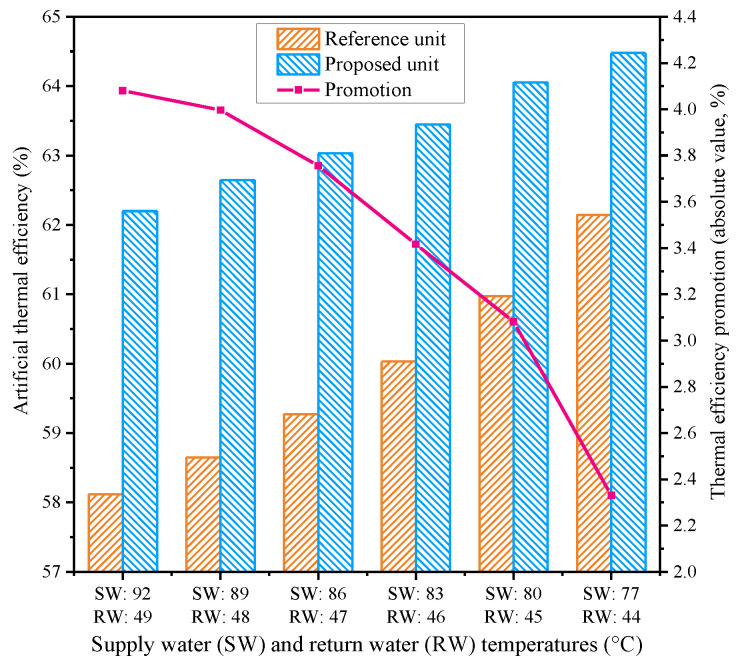
Influences of the supply water and return water temperatures on the performances of the reference and proposed units.

When the demand of the heating service changes, the supply water and return water temperatures of the primary loop vary at the same time, according to the actual district heating condition, and are positively related to each other. To explore how the new design enhances the unit performance under different supply water and return water temperatures, several representative supply water and return water temperatures were selected for comparative study based on the actual operation data of the reference unit during the heating season, and the results are depicted in Figure 10. With the decreases of the supply water and return water temperatures, the artificial thermal efficiencies of the two units grow, but the thermal efficiency promotion diminishes. These results can be explained by noting that when the supply water and return water temperatures are relatively high, more extraction steam from the IPT is employed for heating the supply water, resulting in the AT generating more work, which improves unit efficiency.

#### 4.3.4. Effect of Turbine Back Pressure

**Figure 11:**
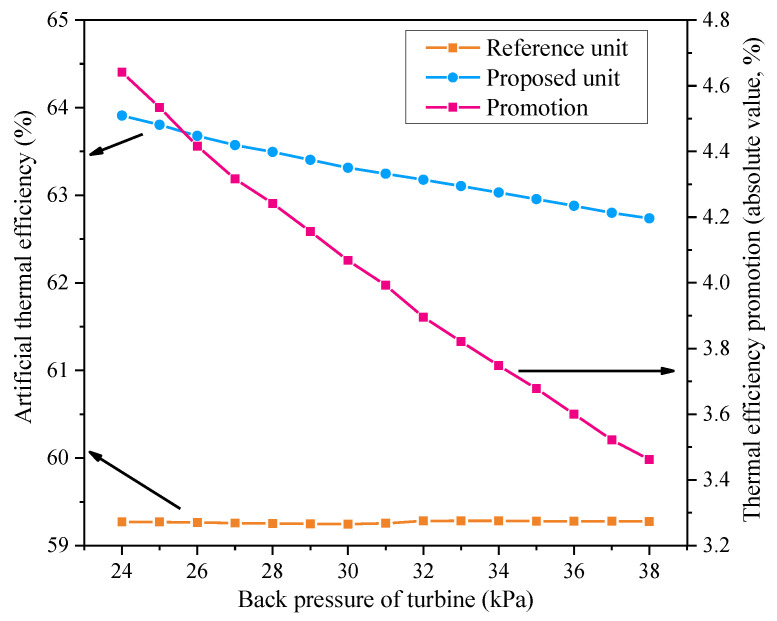
Influences of the turbine back pressure on the performances of the reference and proposed units.

Figure 11 shows how turbine back pressure affects the artificial thermal efficiency of the cogeneration unit with or without the reformative design. These data imply that when the back pressure gets bigger, the artificial thermal efficiency of the reference unit remains almost unchanged, but the artificial thermal efficiency of the modified one drops and the efficiency promotion also declines. This is because the HBP heater can heat the supply water up to a higher temperature under a larger back pressure, leading to less heat being absorbed by the supply water in the ES heater, and therefore less steam being extracted from the IPT. This process reduces the work output of the AT and weakens the energy-saving effect of the proposed concept.

## 5. Techno-Economic Analysis

In the optimized configuration, the additional turbine and generator are implemented, which incurs extra cost. The capital investments ($C_{\mathrm{CI}}$, million USD) of the extra equipment were estimated according to [43,44], and the relations used are mentioned in Table 6.

The energy-saving benefits of the suggested retrofitting in different months of the heating season were derived for economic analysis, as shown in Table 7. These data provide evidence that the reformative scheme can significantly improve the unit efficiency in each period of the heating season, which can create accessorial income by generating more electricity and therefore greater economic revenue. As such, the net annual earning of the new design (*C*_NAE_, million USD) can be obtained as follows:

(8) $$C_{\mathrm{NAE}}=\sum \Delta P_{\mathrm{net}}\times c_{e}\times N/10^{6}-C_{O\&M}$$

where $c_{e}$ donates the on-grid power tariff, which was set as 55.465 USD/MWh [45]; *N* donates the plant operation hours in one heating season; and $C_{O\&M}$ donates the operation and maintenance costs (million USD), which are 4% of $\sum C_{\mathrm{CI}}$ [46].

In this study, the dynamic payback period (*DPP*, year) and net present value (*NPV*, million USD) have been exploited to assess the economic performance of the reformative configuration [45,47]. The dynamic payback period is the time that the financial return equals the initial capital investment, which means that a shorter payback period indicates a better profitability of the retrofitting. The dynamic payback period can be obtained as

(9) $$\sum_{t=1}^{DDP}\frac{C_{\mathrm{NAE}}}{\left(1+i\right)}-\sum C_{\mathrm{CI}}=0$$

where *i* donates the discount rate, which is regarded as 8% [26].

The net present value represents the accumulated net cash flow of the whole usage period of the modified system, which is formulated as

(10) $$NPV=\sum_{t=1}^{n}\frac{C_{\mathrm{NAE}}}{\left(1+i\right)}-\sum C_{\mathrm{CI}}$$

where *n* donates the plant life time, chosen as 30 years.

Using the models above, the techno-economic evaluation was conducted to examine the applicability of the proposed concept, with the results listed in Table 8. The dynamic payback period of the optimized scheme is 2.82 years, and the net present value reaches 22.76 million USD for the plant life time. Hence, the proposed HBP heating system is economically suitable.

## 6. Conclusions

A modified HBP heating design with energy cascade utilization was proposed to promote the efficiency of CHP and its practicability. A case study of a classic coal-fired subcritical cogeneration unit incorporating the optimized configuration was conducted and compared to the reference case with a conventional HBP heating system. The results of the thermodynamic evaluation indicate that the proposed unit can import 8.4 MW extra electricity with a corresponding energy utilization factor improvement of 1.08 percentage points and artificial thermal efficiency improvement of 2.01 percentage points. The energy conservation mechanism of the proposed concept was revealed based on the first and second laws of thermodynamics. It is found that the exergy efficiency of the supply water heating process is raised by 14.65 percentage points due to the reformative configuration. The sensitivity analysis results show that when the unit heat output rises, and the supply water and return water temperatures and the turbine back pressure drop, the unit thermal efficiency promotion caused by the new design will increase significantly. Moreover, the dynamic payback period of the optimized system is 2.82 years, and the net present value reaches as high as 22.76 million USD, which implies that the proposed scheme is economically viable in engineering. This work provides a broadly applicable and feasible approach to further advance CHP.

## Figures and Tables

**Table 1:** Basic parameters of the reference HBP–CHP unit under the rated heating condition.

Item		Unit	Value
Coal consumption rate		t/h	165.3
Net calorific value of coal		MJ/kg	17.00
Main steam	Flow rate	t/h	986.0
	Pressure	MPa	16.67
	Temperature	°C	537
Reheated steam	Flow rate	t/h	836.2
	Pressure	MPa	3.25
	Temperature	°C	537
Exhaust steam	Total flow rate	t/h	509.1
	Flow rate into HBP heater	t/h	274.4
	Pressure	kPa	34
	Temperature	°C	72
Extraction steam for heating	Flow rate	t/h	167.5
	Pressure	MPa	0.39
	Temperature	°C	247.6
Supply water temperature of primary loop		°C	86
Return water temperature of primary loop		°C	47
Net heat output		MW	300.0
Gross power output		MW	270.0
Auxiliary power ratio		%	8.00
Net power output		MW	248.4

**Table 2:** Parameters of the heat regeneration system of the reference HBP–CHP unit under the rated heating condition.

Item	RH1	RH2	RH3	DEA	RH5	RH6	RH7
Extraction steam temperature (°C)	388.1	320.1	453.8	374.5	247.2	184.4	125.2
Extraction steam pressure (MPa)	5.64	3.50	1.78	0.97	0.37	0.20	0.11
Extraction steam flow rate (t/h)	69.7	70.3	34.7	52.0	27.2	23.2	25.8
Inlet feedwater temperature (°C)	242.6	206.6	182.6	137.7	117.1	99.1	78.6
Outlet feedwater temperature (°C)	273.2	242.6	206.6	178.7	137.7	117.1	99.1
Outlet feedwater pressure (MPa)	16.67	16.68	16.69	0.97	0.97	0.98	0.99
Outlet feedwater flow rate (t/h)	986.0	986.0	986.0	986.0	759.3	759.3	759.3
Drain water temperature (°C)	271.5	242.6	206.6	-	140.5	119.9	101.9

**Table 3:** Model details of the cogeneration unit in Ebsilon Professional software.

Major Component	Module	Details
Boiler	Steam generator	The boiler efficiency is 0.93.
Turbines	Steam turbine	The isentropic efficiencies of the HPT, IPT and LPT are determined by the heat balance diagrams of the reference unit, which range from 0.78 to 0.93. The isentropic efficiency of the AT is chosen as 0.75. Mechanical efficiencies are 0.998.
Electric generators	Generator	The efficiency of the main generator is 0.99. The efficiency of the additional generator is 0.92.
RHs	Feedwater preheater, aftercooler and deaerator	The upper-terminal temperature difference of each feedwater preheater and the lower-terminal temperature difference of each aftercooler are to be specified. The pressure loss is 3%–5% for the steam extraction at different stages. The heat loss is neglected.
CON	Condenser	The upper-terminal temperature difference is 5 °C. The pressure loss of the cooling medium is 50 kPa.
Pumps	Pump	The isentropic efficiencies are 0.80. The mechanical efficiencies are 0.998.
HBP heater and ES heater	Universal heat exchanger	The upper-terminal temperature difference of the HBP heater is 2 °C. The upper-terminal temperature difference of the ES heater is 10 °C.

**Table 4:** Unit gross power output calculation errors of the simulation model under typical conditions. (THA, turbine heat acceptance).

Condition	Design Value (MW)	Calculated Value (MW)	Relative Error (%)
THA	330.02	330.32	+0.09
75% THA	247.54	248.21	+0.27
50% THA	165.02	165.56	+0.33
40% THA	132.03	132.65	+0.47
30% THA	99.02	99.53	+0.52

**Table 5:** Overall performances of the reference and proposed units.

Item		Reference Unit	Proposed Unit	Difference
Total energy of fuel input (MW)		780.5	780.5	0
Extraction steam at ES heater inlet	Flow rate (t/h)	167.5	183.0	+15.5
	Pressure (MPa)	0.39	0.09	−0.30
	Temperature (°C)	247.6	129.1	−118.5
	Specific enthalpy (kJ/kg)	2959.9	2735.8	−224.1
	Saturation temperature (°C)	142.7	96.7	−46.0
Condensate at ES heater outlet	Pressure (MPa)	0.39	0.09	−0.30
	Temperature (°C)	75.0	75.0	0
	Specific enthalpy (kJ/kg)	314.2	314.0	−0.2
Exhaust steam	Total flow rate (t/h)	509.1	494.0	−15.1
	Flow rate into HBP heater (t/h)	274.4	274.4	0
	Flow rate into CON (t/h)	234.7	219.6	−15.1
	Energy loss in CON (MW)	144.3	135.3	−9.0
	Recovery efficiency (%)	53.90	55.49	+1.59
Net heat output (MW)		300.0	300.0	0
Net power output attributed to HPT, IPT and LPT (MW)		248.4	247.9	−0.5
Net power output attributed to AT (MW)		0	8.9	+8.9
Total net power output (MW)		248.4	256.8	+8.4
Energy utilization factor (%)		70.26	71.34	+1.08
Artificial thermal efficiency (%)		59.27	61.28	+2.01

**Table 6:** Capital investment calculations of the additional equipment in the proposed unit.

Equipment	Unit	Capital Investment
Additional turbine	million USD	$C_{\mathrm{CI},\mathrm{st}}=6000\times {\left(P_{\mathrm{nom}}\times 10^{3}\right)}^{0.7}/10^{6}$
Additional generator	million USD	$C_{\mathrm{CI},g}=60\times {\left(P_{\mathrm{nom}}\times 10^{3}\right)}^{0.95}/10^{6}$

**Table 7:** Thermal performances of the reference and proposed units for each month during the heating season.

Item		Nov.	Dec.	Jan.	Feb.	Mar.
Heating time (day)		15	31	31	28	15
Net heat output (MW)		173.5	271.3	309.7	304.4	198.7
Supply water temperature (°C)		77	83	89	86	80
Return water temperature (°C)		44	46	48	47	45
Reference unit	Net power output (MW)	164.1	212.2	251.8	245.7	168.5
	Energy utilization factor (%)	65.41	72.55	70.52	71.16	68.94
	Artificial thermal efficiency (%)	53.43	62.49	59.52	60.47	57.46
Proposed unit	Net power output (MW)	167.1	219.1	261.7	254.3	172.7
	Net power output increment (MW)	2.9	7.0	9.9	8.6	4.1
	Energy utilization factor (%)	65.97	73.62	71.82	72.31	69.73
	Artificial thermal efficiency (%)	54.39	64.61	61.97	62.67	58.91

**Table 8:** General economic analysis results of the proposed scheme.

Item	Unit	Value
Total capital investment ($\sum C_{\mathrm{CI}}$)	million USD	4.07
Annual operation and maintenance cost ($C_{O\&M}$)	million USD	0.16
Net annual earning ($C_{\mathrm{NAE}}$)	million USD	2.02
Dynamic payback period (*DPP*)	year	2.82
Net present value (*NPV*)	million USD	22.76

## Nomenclature

***Abbreviations***			
AHE	absorption heat exchange	FWP	feed water pump
AHP	absorption heat pump	HBP	high back pressure
AT	additional turbine	HPT	high-pressure turbine
CHP	combined heat and power	IPT	intermediate-pressure turbine
CON	condenser	LPT	low-pressure turbine
CP	condensate pump	RH	regenerative heater
DEA	deaerator	RW	return water
EUD	energy utilization diagram	SW	supply water
ES	extraction steam	THA	turbine heat acceptance
ET	exhaust steam		
***Symbols***			
Δ	difference	*P*	power, MW
*η*	efficiency	*Q*	heat, MW or kJ
*A*	energy level	*S*	entropy, kJ/K
*ATE*	artificial thermal efficiency	*T*	temperature, K
*C*	cash flow, million USD	*c*	electricity price, USD/MWh
*DDP*	dynamic payback period, year	*ex*	specific exergy, kJ/kg
*EUF*	energy utilization factor	*h*	specific enthalpy, kJ/kg
*EX*	exergy, MW	*i*	discount rate
*N*	plant operation hours	*m*	mass flow rate, kg/s
*NPV*	net present value, million USD	*s*	specific entropy, kJ/kgK
***Subscripts***			
0	environmental state	g	generator
CI	capital investment	h	heating
NAE	net annual earning	hot	hot fluid
O&M	operation and maintenance	in	inlet
b	boiler	net	net value
cold	cold fluid	m	matter
e	electricity	nom	nominal
es	extraction steam	out	outlet
et	exhaust steam	rec	recovery
ex	exergy	ref	reference
f	fuel		
